# Exploring Regularization Methods for Domain Generalization in Accelerometer-Based Human Activity Recognition

**DOI:** 10.3390/s23146511

**Published:** 2023-07-19

**Authors:** Nuno Bento, Joana Rebelo, André V. Carreiro, François Ravache, Marília Barandas

**Affiliations:** 1 Associação Fraunhofer Portugal Research, Rua Alfredo Allen 455/461, 4200-135 Porto, Portugal; 2 ICOM France, 1 Rue Brindejonc des Moulinais, 31500 Toulouse, France

**Keywords:** Human Activity Recognition, deep learning, Domain Generalization, regularization, accelerometer

## Abstract

The study of Domain Generalization (DG) has gained considerable momentum in the Machine Learning (ML) field. Human Activity Recognition (HAR) inherently encompasses diverse domains (e.g., users, devices, or datasets), rendering it an ideal testbed for exploring Domain Generalization. Building upon recent work, this paper investigates the application of regularization methods to bridge the generalization gap between traditional models based on handcrafted features and deep neural networks. We apply various regularizers, including sparse training, Mixup, Distributionally Robust Optimization (DRO), and Sharpness-Aware Minimization (SAM), to deep learning models and assess their performance in Out-of-Distribution (OOD) settings across multiple domains using homogenized public datasets. Our results show that Mixup and SAM are the best-performing regularizers. However, they are unable to match the performance of models based on handcrafted features. This suggests that while regularization techniques can improve OOD robustness to some extent, handcrafted features remain superior for domain generalization in HAR tasks.

## 1. Introduction

Human Activity Recognition (HAR) addresses the problem of identifying specific kinds of physical activities or movements performed by a person based on data that can be collected by several types of sensors [1]. It is a critical technology that supports several applications, including remote patient monitoring, locomotor rehabilitation, security, and pedestrian navigation [2]. This work focuses on HAR relying on inertial sensors, such as accelerometers, which measure the acceleration of a body, or gyroscopes, which measure angular velocity. These sensors are usually combined in Inertial Measurement Units (IMUs), which are present in most smartphones and smartwatches, and nearly ubiquitous in our daily life [3]. This translates into an increasing availability of sensor data, which, along with its importance in several fields, has motivated the growth of HAR in the past years [1].

Despite being a widely studied field, there are still challenges to be faced in HAR, one of which is the difficulty in developing models that generalize effectively across different domains [4]. This results in HAR models that perform well when tested on a randomly selected portion of a meticulously acquired dataset, but exhibit a performance decline when tested in realistic Out-of-Distribution (OOD) settings. These settings are characterized by a domain shift (or distribution shift) between the source and target domains [5]. In the HAR context, this can occur when the models are tested across different users, devices, sensor positions, or data acquisition setups [6,7,8].

The problem of distribution shift can be found in most data-related fields. A straightforward solution involves collecting data from the target domain and adapting the model, which was initially trained on source data, using the target data. This approach, known as domain adaptation, has been extensively explored [5]. However, it presupposes the availability of target data, a condition that may not always be met in real-world scenarios. In order to simultaneously tackle the domain shift and the absence of target data, the problem of domain generalization originated. Domain Generalization (DG) focuses on leveraging only source data to develop models that generalize to OOD target domains [5].

In traditional HAR approaches, features are extracted manually through signal processing techniques before being used as input to a machine learning model [9]. More recently, deep learning has attracted attention as a potential tool for HAR tasks [10]. In this modern approach, features are automatically extracted during the training process [10]. Given the large number of learnable parameters associated with deep learning models, they should be able to learn more complex and discriminative features [11]. This capability is expected to help deep learning thrive in DG scenarios. Nevertheless, several limitations have been identified upon deploying deep learning models, such as the convergence to solutions that rely on spurious correlations [12]. In our previous work, Bento et al. [13] compared the effectiveness of Handcrafted (HC) features versus deep neural representations for DG in HAR. Our findings revealed that while deep learning models initially outperformed those based on HC features, this trend was reversed as the distance from the training distribution increased, creating a gap between these methods in the OOD regime.

Our work attempts to bridge this gap by using regularization, which primarily focuses on mitigating overfitting, consequently leading to improved generalization performance [14,15]. For that purpose, several regularization methods are compared by following a methodology introduced in Bento et al. [13], leveraging five public datasets that are homogenized, so that they can be arranged in different combinations, creating multiple OOD settings.

The research questions addressed by this work are the following:

1. How do different regularization methods impact the Domain Generalization performance of human activity recognition models?
2. Can regularization methods bridge the OOD performance gap between deep neural networks and models based on HC features?

## 2. Related Work

Concerning classical machine learning approaches using HC features, several algorithms have been proposed for the recognition of human activities. Despite the considerable progress made by these algorithms in HAR, they may not capture more complex signal patterns, which can hinder their generalization performance [10]. To overcome this limitation, research has turned to Deep Learning (DL) models, which can automatically extract high-level features from raw data [10].

With that in mind, recent work [16,17,18,19,20] has compared traditional Machine Learning (ML) with DL approaches for HAR. Their findings consistently demonstrate that deep learning outperforms traditional methods. However, it should be noted that, in these experiments, the data splits were created by randomly shuffling the datasets. As a result, the training and test sets contain samples from distinct domains, therefore mitigating the distribution shift in their evaluations. As such, models optimized for these data splits may achieve suboptimal results in a real-world environment [21].

In studies where data splits took into account the distribution shift caused by different domains [13,20,22,23], the ability of DL methods to generalize has been put into question, as traditional ML models achieved similar or even better results, in some cases.

One of the reasons why DL models may not generalize well is that they are known to suffer from overfitting since they possess many parameters and their optimization process is not perfect [14]. One of the ways to prevent overfitting is by using regularization methods, which can be seen as applying constraints to the training process or the models in the form of penalties applied to parameter norms (e.g., $L_{2}$ regularization), elimination of parameters (e.g., dropout), early stopping, among other techniques [14]. As well as these popular regularization techniques, recent work has yielded progressively superior methods [24,25,26,27]. Mixup regularization works by performing a linear interpolation between input/target pairs and has been shown to outperform previous methods such as dropout and weight decay [24,25]. The optimization algorithms used for training can also be considered a form of regularization [28]. Methods that attempt to regularize stochastic gradient descent include averaging weights over various iterations [29] or actively searching for flat minima [26]. Sparsity is another form of regularization that can improve both generalization performance and model efficiency in deep learning by promoting the use of fewer non-zero parameters, leading to simpler models [30]. Sparse training is an efficient and effective way to add this type of regularization to a neural network [27,30,31].

Distributionally Robust Optimization (DRO) is a promising approach for addressing the need for optimizing models for Domain Generalization [21,32]. These methods usually regularize the training process by considering the distribution shift between the existing domains. Invariant Risk Minimization (IRM) [21] and Variance—Risk Extrapolation (V-REx) [33] introduce penalties to the loss function with the objective of learning representations that are invariant across multiple domains. Ahuja et al. [34] showed that adding a penalty based on the information bottleneck principle to IRM improves generalization—IB-IRM.

Some of the aforementioned regularization methods have been investigated as a potential solution to the OOD generalization problem in HAR. Gagnon et al. [35] included a HAR dataset in their Domain Generalization benchmark. Their results indicate a 9.07% drop in accuracy from 93.35% In-Distribution (ID) to 84.28% OOD on a dataset where different devices worn in different positions characterize the possible domains. IB-IRM [34] was the best-performing method. However, results did not improve significantly over empirical risk minimization (ERM), which is still a strong baseline [36]. Lu et al. [37] introduced a semantic-aware version of Mixup, which outperformed several Domain Generalization methods in HAR tasks. They presented results across different users, datasets, and positions. However, handcrafted features were not addressed in their work. Trabelsi et al. [20] compared three deep learning approaches and a random forest classifier with handcrafted features as input. Similarly to the experiments in our work, the datasets were homogenized by including only shared activities and separating the test sets by user. They concluded that only one of the deep learning approaches outperformed the baseline model with handcrafted features. Regularization methods were not studied in their work.

Our previous work, Bento et al. [13] showed that while DL methods outperformed traditional ML approaches when the training and test sets were split randomly, as the distance between the distributions grows, the tendency inverts, with methods based on DL usually performing worse in OOD settings. This paper builds on that work, adding different regularization methods to the models in order to assess if and by how much the OOD performance gap between HC features and deep representations is reduced. Our experiments include four Domain Generalization settings with different distances between training and test sets. To the best of our knowledge, this is the first attempt at comparing regularization methods for Domain Generalization in HAR.

## 3. Methodology

### 3.1. Datasets

The data employed in this study are the same as that used in Bento et al. [13]. Therefore, the datasets are composed of human activity data collected with smartphones and wearable IMUs. All the datasets are publicly available, and a comprehensive description of each is presented in Table 1.

The datasets were selected according to three criteria: (a) a sampling frequency superior or equal to 50 Hz; (b) most of the main human activities in the literature (walking, sitting, standing, running, upstairs, and downstairs); and (c) at least one common sensor position with another of the chosen datasets.

For this study, only the accelerometer data were used. In addition to the three accelerometer channels (x, y, and z) produced directly by the sensors, a fourth channel comprising the accelerometer magnitude was computed and utilized in the classification process. Five-second windows without overlap were then extracted from those four channels.

Data homogenization consisted of resampling all the datasets to 50 Hz and mapping activity labels of all datasets to a shared naming convention: “walking”, “running”, “sitting”, “standing”, and “stairs”. Note that the class “stairs” does not differentiate between ascending and descending stairs. Given the discrepancy between the number of windows generated by each dataset, only one-third of the windows from the RealWorld dataset were randomly sampled and used in the experiments. Table 2 displays the final distribution of windows and activities for each dataset.

For further details regarding the datasets used and decisions concerning data preprocessing, please refer to Bento et al. [13].

### 3.2. Handcrafted Features

The TSFEL library [45] was used to extract features from the windows produced from each public dataset. Features including individual coefficients and audio-related features were excluded to reduce the computation time. This resulted in a total of 192 features per window.

The following steps were used to split the samples based on their task (see Section 4) and perform Z-score normalization with statistical information regarding the training set. The classification algorithms used were a Logistic Regression (LR) and a Multilayer Perceptron (MLP).

Additional details regarding feature extraction and preprocessing can be found in Bento et al. [13].

### 3.3. Deep Learning

The architectures used in our experiments were different variations of convolutional neural networks. We chose the two best-performing architectures from [13], which were CNN-base and ResNet. Refer to the original paper for a detailed explanation of the used architectures and training process.

**Figure 1:**
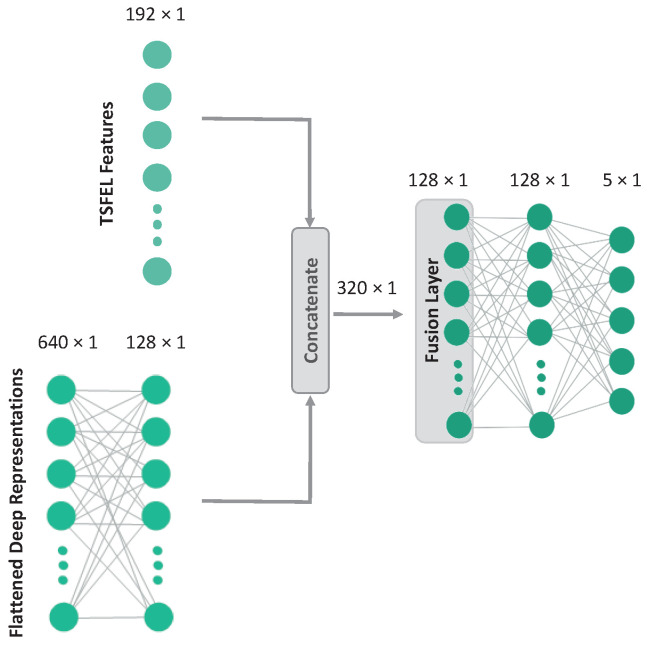
Simplified illustration of the hybrid version of CNN-base (excluding the CNN backbone for ease of visualization) [13].

For the hybrid models, the HC features are concatenated with the flattened representations of each model and fed to a fusion layer before entering the final classification layer. An illustration of the hybrid version of CNN-base is shown in Figure 1.

For all these models, the input windows were scaled by Z-score normalization, with mean and standard deviation computed across all the windows of the train set.

### 3.4. Regularization

In this study, several regularization methods are compared:

- Mixup regularization [24,25]: It linearly interpolates input/target pairs to create new examples, which are used to make decision boundaries smoother and avoid overfitting.
- Sharpness-Aware Minimization (SAM) [26]: Optimization method that actively seeks flat minima. This type of minima was shown to be less prone to overfitting [46].
- GraNet [27]: It is a state-of-the-art method for sparse training that gradually reduces the number of non-zero weights during training.
- IRM [21]: It attempts to learn invariant representations by minimizing the sum of the squared norms of the gradients across multiple environments.
- V-REx [33]: It has the same purpose of IRM, but instead it minimizes the gradient variance across environments.
- IB-IRM [34]: It introduces a term to the IRM loss corresponding to the variance in the model parameters, following the information bottleneck principle.

### 3.5. Evaluation

Various metrics are used in research literature to assess model performance. These include accuracy, sensitivity, specificity, precision, recall, and f1-score [2]. However, due to the frequent occurrence of class imbalance in many public HAR datasets (as indicated in Table 2), f1-score was chosen as the primary performance metric, as it proved to be more resilient than accuracy in these types of situations [47]. For the sake of comparing deep learning models and traditional models using HC features, f1-scores were used as the comparison metric. This comparison was carried out across multiple OOD scenarios and took into consideration five public HAR datasets.

## 4. Experiments and Results

**Figure 2:**
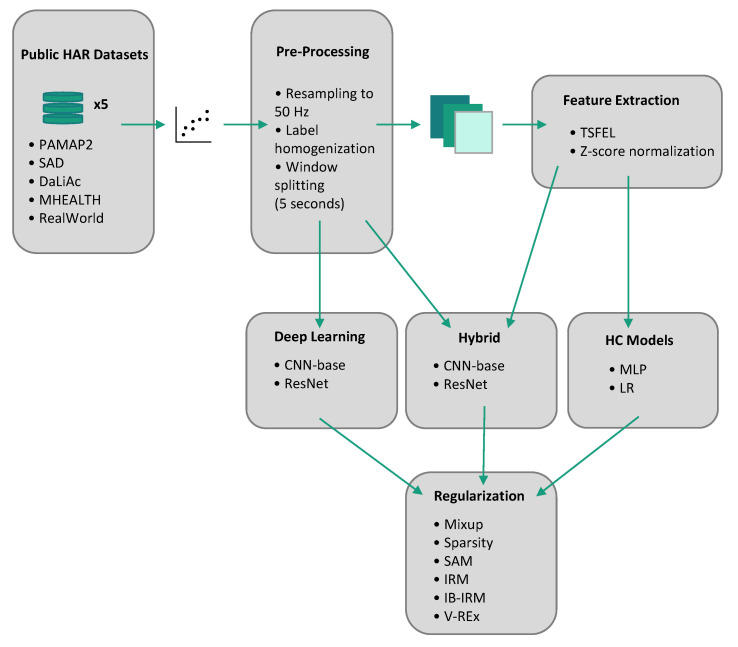
Scheme of the experimental pipeline.

The goal of this study is to assess the improvement brought by using different regularization techniques on Domain Generalization tasks involving models based on HC features and deep neural networks. To that end, various combinations of model architectures and regularization methods were implemented and evaluated. A scheme of the full pipeline used for the experiments is presented in Figure 2.

HAR is a task where different domains naturally occur, becoming a Domain Generalization task if these domains are preserved when splitting the data into training and test sets. To measure how different a test set is from the training set, or how OOD it is, Bento et al. [13] computed Wasserstein distance ratios. Following that study, our experiments were conducted over the same four domain generalization settings, comprised of a baseline ID setting and three OOD settings [13]: (a) splitting by user within the same dataset (OOD-U); (b) leaving a dataset out for testing (OOD-MD); and (c) training on a dataset and leaving another for testing (OOD-SD). Test sets in the OOD-U setting were closer to the training distribution, being further away in the OOD-MD and OOD-SD settings. Non-exhaustive hyperparameter optimization was applied to the regularization methods on a small private HAR dataset. The chosen hyperparameters for each method are specified in Table 3.

Table 4 presents the results for the first experiment, which combines neural network architectures and regularizers. The DRO models (IRM, V-REx, and IB-IRM) used in this experiment require the formulation of different environments (i.e., domains) in the training set. For the ID and OOD-U settings, the environments are split by the user. For OOD-MD, each environment can be trivially devised as a dataset. However, for the OOD-SD, only a single dataset is present in the training set, so there is no trivial way to simulate the distribution shift that occurs between the training and test sets. Consequently, this setting was removed from the experiment. In Table 4, it can be verified that ResNet is the best-performing deep learning architecture, as it consistently shows higher f1-scores compared to CNN-base. For CNN-base, only SAM improved over the baseline model without regularization. Still, this improvement was not significant, and the performance was far from its hybrid version (CNN-base hybrid). For the ResNet architecture, Mixup, SAM, IRM, and IB-IRM improved over the baseline. Mixup achieved the best results (76.44%), marginally surpassing the score of the hybrid version (76.29%). Overall, Mixup and SAM can be considered the best-performing regularizers since the scores either improved or remained approximately the same in both architectures. The larger improvement verified on the ResNet may be due to the increased effectiveness of regularization methods in overparameterized regimes [12,48].

DRO methods are known to heavily depend on the chosen hyperparameters [36]. This may have hindered their performance. For these methods, hyperparameter optimization was performed over only three different values (10, 100, and 1000) for their regularization weights since the regularization methods should require as little computational overhead as possible.

As a second experiment, the best architecture (ResNet) and the two best regularizers (Mixup and SAM) were chosen, so that it could be assessed whether a combination of the best regularizers could further improve the generalization of deep learning models. The results are presented in Table 5. Since none of the DRO methods were considered, the OOD-SD setting could be recovered for this experiment, as none of the remaining methods require information about the environments. After adding the OOD-SD setting, the ResNet hybrid (66.77%) slightly outperformed the regularized ResNet models (66.42% and 66.48%). However, the difference is minimal, so we can consider that their performance is approximately the same. In the OOD-MD setting, ResNet with Mixup regularization (71.18%) outperformed some classical models. Despite that, this improvement loses significance when assessing the average scores.

The best-performing model across both experiments was TSFEL + LR, followed by the remaining HC feature-based models, which includes TSFEL + MLP and the hybrid models. Despite the effectiveness of regulariztion, it was insufficient for deep learning models to reach the desired OOD performance levels. Still, as regularizers did not improve the overall results of any of the models that make use of HC features (i.e., TSFEL + LR, TSFEL + MLP and hybrid models), it can be observed that these regularizers can help bridge the generalization gap between deep learning models and models based on HC features.

## 5. Discussion

The work conducted in this study evaluated the differences in the performance of different regularization techniques on Domain Generalization tasks applied to HAR classification.

In the first experiment, a comparison between various combinations of neural network architectures and regularizers was carried out. ResNet outperformed CNN-base consistently across different ID and OOD settings. This result aligns with the general perception about the superiority of ResNet in handling a broad range of tasks due to its deeper architecture and residual connections, enabling it to learn more complex representations [49]. Regarding regularization methods, Mixup and SAM were considered to be the best-performing methods in both architectures. This agrees with the original premises behind these techniques, as Mixup attempts to improve generalization by enforcing a smoother decision boundary [24], while SAM adds robustness to label noise [26].

In the second experiment, a combination of the two best regularizers (Mixup and SAM) and the best architecture (ResNet) was performed to assess if it further improves the generalization of deep learning models. Combining the two methods did not yield better results than using Mixup alone. This experiment also showed that regularization only improved the performance of deep learning models that did not include HC features. The fact that the average OOD score of the ResNet hybrid did not change with the use of regularizers may indicate that the use of these features as an auxiliary input can already have a regularizing effect.

Overall, despite none of the deep learning models being able to surpass the performance of models based on HC features, regularization improved the generalization ability of deep Llearning models and was as effective as the auxiliary features for the ResNet architecture. Moreover, as regularizers did not improve the results for the classical methods, they were clearly able to reduce the generalization gap.

This study shows that HC features still have their place in modern machine learning, as they can be more robust to distribution shift and allow simple classifiers to achieve better results.

We also showed that merging all the datasets for training (OOD-MD) resulted in a performance gain of 20% when compared to using a single dataset for training (OOD-SD). This indicates that each dataset has limited information. In practice, if a larger space of possible scenarios can be covered during the acquisition process, it will result in a more diverse dataset and, consequently, in better generalization. This means that an ML-based HAR system can improve if data are recorded using a wider range of devices, users, sensor positions, and physical environments, among other possible factors of variation.

Our work has some limitations, as the choice of datasets was limited to the field of HAR and only a few regularization techniques were tested. Additional research should explore datasets from different fields and a wider range of increasingly novel regularization methods to comprehensively understand their effects on domain generalization. Future work could also investigate the use of different neural network architectures, such as transformers, or even neural architecture search [50] since it has been shown that, in some cases, the choice of model architecture may have more impact than the loss function [51]. Domain-specific regularization methods [37] were also demonstrated to have the potential to vastly improve the generalization of deep learning models. Despite that, these methods suffer from the caveat of not being directly applicable to other tasks.

## 6. Conclusions

This study has addressed the impact of different regularization methods on the domain generalization performance of HAR models and whether these methods can bridge the OOD performance gap between deep neural networks and HC feature-based models. Our experimental results indicate that state-of-the-art regularization methods, such as Mixup and SAM, can improve OOD generalization and reach the results of hybrid models. However, it was not enough to be on par with classical ML approaches. We conclude that the use of HC features, regularizers, and diverse training data may enable more robust HAR systems.

Overall, this study contributes to the understanding of how regularization methods can impact the Domain Generalization performance of HAR models and their potential to narrow the OOD performance gap between deep neural networks and traditional approaches. These insights will be valuable for researchers and practitioners working on HAR and related fields, helping them build more robust and generalizable models for real-world applications.

## Figures and Tables

**Table 1:** Description of the datasets, including number of subjects, activities, devices, sample rate, positions, and sources.

Dataset	Subjects	Activities	Devices	Sample Rate	Positions	Source
PAMAP2	9	Sitting, lying, standing, walking, ascending stairs, descending stairs, running.	3 IMUs	100 Hz	Wrist, chest, and ankle.	[38,39]
SAD	10	Sitting, standing, walking, ascending stairs, descending stairs, running and biking.	5 smartphones	50 Hz	Jeans pocket, arm, wrist, and belt.	[40]
DaLiAc	19	Sitting, lying, standing, walking outside, ascending stairs, descending stairs, treadmill running.	4 IMUs	200 Hz	Hip, chest, and ankles.	[41]
MHEALTH	10	Sitting, lying, standing, walking, climbing/descending stairs, jogging, running.	3 IMUs	50 Hz	Chest, wrist, and ankle.	[42,43]
RealWorld	15	Sitting, lying, standing, walking, ascending stairs, descending stairs, running/jogging.	6 IMUs	50 Hz	Chest, forearm, head, shin, thigh, upper arm, and waist.	[44]

**Table 2:** Distribution of samples and activity labels per dataset. The # symbol represents the number of samples. Retrieved from [13].

	Activity	Datasets (%)					Total	
		**PAMAP2**	**SAD**	**DaLiAc**	**MHEALTH**	**Real World**	**%**	**#**
	Run	10.5	16.9	20.0	33.3	19.1	18.3	7975
	Sit	19.8	16.9	10.6	16.7	17.0	16.3	7102
	Stairs	23.6	32.2	12.3	16.7	30.0	26.3	11,460
	Stand	20.4	16.9	10.6	16.7	16.4	16.2	7047
	Walk	25.7	16.9	46.5	16.7	17.5	22.8	9927
**Total**	**%**	12.7	24.4	15.3	4.96	42.6	-	-
	**#**	5541	10,620	6644	2160	18,546	-	43,511

**Table 3:** Chosen hyperparameters for each regularization method.

Method	Hyperparameter	Value
Mixup	α	0.1
GraNet	prune rate	0.5
	initial density	0.5
	final density	0.1
SAM	base optimizer	Adam
	ρ	0.05
IRM	λ	100
V-REx	β	10
IB-IRM	λ	100
	γ	10

**Table 4:** Average f1-score in percentage (%) over all the tasks in all the settings except OOD-SD. Values in bold indicate the best performance for each setting. * indicates the experiments without regularization performed in [13].

Model	Setting			Avg. OOD
	ID	OOD-U	OOD-MD	
CNN-base *	92.10±5.06	80.79±9.68	66.94±5.19	73.87±5.49
CNN-base Mixup	91.18±4.56	80.66±9.33	67.05±3.88	73.86±5.05
CNN-base Sparse	92.01±5.02	80.75±10.04	66.11±5.48	73.43±5.72
CNN-base SAM	91.44±4.86	81.46±10.56	66.68±4.49	74.07±5.74
CNN-base IRM	90.70±5.41	80.24±9.89	66.86±4.25	73.55±5.38
CNN-base V-REx	88.69±7.29	80.31±9.71	67.05±5.68	73.68±5.63
CNN-base IB-IRM	84.33±14.98	80.55±10.60	65.59±4.90	73.07±5.84
CNN-base hybrid *	93.48±4.35	85.28±6.64	67.74±3.37	76.51±3.72
ResNet *	92.46±4.73	81.16±9.60	67.22±4.89	74.19±5.39
ResNet Mixup	92.10±4.59	81.71±9.91	71.18±4.18	76.44±5.38
ResNet Sparse	92.48±4.61	80.86±10.80	67.10±3.32	73.98±5.65
ResNet SAM	92.01±4.46	81.36±10.56	68.82±3.57	75.09±5.57
ResNet IRM	91.40±4.68	80.66±9.80	69.00±5.53	74.83±5.63
ResNet V-REx	90.64±5.54	80.74±9.61	67.00±5.66	73.87±5.58
ResNet IB-IRM	85.14±15.26	80.63±10.00	68.69±5.52	74.66±5.71
ResNet hybrid *	93.79±4.21	84.71±7.72	67.87±3.40	76.29±4.22
TSFEL + MLP *	92.87±4.70	87.09±5.35	70.11±3.57	78.60±3.22
TSFEL + LR *	90.54±5.15	87.08±5.55	71.94±3.19	79.51±3.20

**Table 5:** Average f1-score in percentage over all the tasks in a given setting. Values in bold indicate the best performance for each setting. * indicates the experiments without regularization performed in [13].

Model	Setting				Avg. OOD
	ID	OOD-U	OOD-MD	OOD-SD	
ResNet *	92.46±4.73	81.16±9.60	67.22±4.89	46.57±4.84	64.98±3.94
ResNet Mixup	92.10±4.59	81.71±9.91	71.18±4.18	46.56±6.27	66.48±4.15
ResNet Mixup SAM	92.03±4.27	82.22±10.32	70.04±4.00	46.99±6.25	66.42±4.24
ResNet hybrid *	93.79±4.21	84.71±7.72	67.87±3.40	47.73±2.11	66.77±2.90
ResNet hybrid Mixup SAM	93.30±3.54	83.83±9.00	69.87±2.47	46.60±4.22	66.77±3.41
TSFEL + MLP *	92.87±4.70	87.09±5.35	70.11±3.57	51.45±5.31	69.55±2.78
TSFEL + LR *	90.54±5.15	87.08±5.55	71.94±3.19	50.97±3.29	70.00±2.40
TSFEL + LR Mixup SAM	90.38±5.03	87.05±5.23	71.67±3.97	50.41±3.58	69.71±2.49
TSFEL + MLP Mixup SAM	93.03±4.58	87.46±5.97	70.39±2.71	51.26±4.00	69.71±2.56

## Data Availability

Not applicable.
